# The Suitability of Propolis as a Bioactive Component of Biomaterials

**DOI:** 10.3389/fphar.2022.930515

**Published:** 2022-06-08

**Authors:** Ronny Lesmana, Felix Zulhendri, James Fearnley, Ilham A. Irsyam, Renaldi P. H. N. Rasyid, Trimurni Abidin, Rizky Abdulah, Auliya Suwantika, Anant Paradkar, Arief S. Budiman, Timotius Pasang

**Affiliations:** ^1^ Physiology Division, Department of Biomedical Sciences, Faculty of Medicine, Universitas Padjadjaran, Bandung, Indonesia; ^2^ Center of Excellence in Higher Education for Pharmaceutical Care Innovation, Universitas Padjadjaran, Bandung, Indonesia; ^3^ Biological Activity Division, Central Laboratory, Universitas Padjadjaran, Bandung, Indonesia; ^4^ Kebun Efi, Kabanjahe, Indonesia; ^5^ Apiceutical Research Centre, Whitby, United Kingdom; ^6^ Department of Orthopaedics and Traumatology, Faculty of Medicine, Universitas Sumatera Utara, Medan, Indonesia; ^7^ Department of Orthopaedics, Faculty of Medicine, Universitas Padjadjaran, Bandung, Indonesia; ^8^ Department of Conservative Dentistry, Universitas Sumatera Utara, Medan, Indonesia; ^9^ Department of Pharmacology and Clinical Pharmacy, Faculty of Pharmacy, Universitas Padjadjaran, Bandung, Indonesia; ^10^ Centre for Pharmaceutical Engineering Science, School of Pharmacy, University of Bradford, Bradford, United Kingdom; ^11^ Department of Manufacturing and Mechanical Engineering and Technology, Oregon Institute of Technology, Klamath Falls, OR, United States; ^12^ Industrial Engineering Department, BINUS Graduate Program, Bina Nusantara University, Jakarta, Indonesia

**Keywords:** propolis, biomaterial, orthopedics, dentistry, natural product

## Abstract

Propolis is a resinous product collected by bees from plant exudates to protect and maintain hive homeostasis. Propolis has been used therapeutically for centuries as folk medicine. Modern research investigating the diversity of the chemical composition and plant sources, biological activity, extraction processes, analytical methods, and therapeutic properties in clinical settings have been carried out extensively since the 1980s. Due to its antimicrobial, anti-inflammatory, and immuno-modulator properties, propolis appears to be a suitable bioactive component to be incorporated into biomaterials. This review article attempts to analyze the potential application of propolis as a biomaterial component from the available experimental evidence. The efficacy and compabitility of propolis depend upon factors, such as types of extracts and types of biomaterials. Generally, propolis appears to be compatible with hydroxyapatite/calcium phosphate-based biomaterials. Propolis enhances the antimicrobial properties of the resulting composite materials while improving the physicochemical properties. Furthermore, propolis is also compatible with wound/skin dressing biomaterials. Propolis improves the wound healing properties of the biomaterials with no negative effects on the physicochemical properties of the composite biomaterials. However, the effect of propolis on the glass-based biomaterials cannot be generalized. Depending on the concentration, types of extract, and geographical sources of the propolis, the effect on the glass biomaterials can either be an improvement or detrimental in terms of mechanical properties such as compressive strength and shear bond strength. In conclusion, two of the more consistent impacts of propolis across these different types of biomaterials are the enhancement of the antimicrobial and the immune-modulator/anti-inflammatory properties resulting from the combination of propolis and the biomaterials.

## Introduction

Propolis is a resinous beehive-derived product. The word “propolis” comes from the Greek “pro”, which means “before”, and “polis”, meaning city. Before the City or Defender of the City - propolis is the defender of the beehive. One of the oldest pre-historical records of the use of bee products by humans dates back to c. 13,000 BCE. Hippocrates, the father of modern medicine, used propolis to treat wounds (both internal and external) and ulcers. In communities where beekeeping is an integral part of daily life, propolis has been used to treat many ailments because of its wide ranging pharmacological properties such as antimicrobial, antioxidant, anti-inflammatory, and anti-proliferative. In addition, propolis is also considered cost-effective and considerably safe with minimal adverse effects (Kuropatnicki et al., 2013; Bankova et al., 2019a; Popova et al., 2021b). As a result, we believe it worth investigating whether propolis may prove suitable to be incorporated as the bioactive substance in biomaterials. The objective of the present review is to analyze the potential use of propolis as a component of biomaterials.

Bees primarily forage for three plant materials; nectar, pollen, and propolis, in order to survive and maintain the functionality of hives. Nectar which will be converted to honey through dehydration, and pollen serve as the energy/carbohydrate source and protein source, respectively (Wang et al., 2014). Propolis, on the other hand, has a different function in the beehive. Propolis is collected by the bees from plant exudates (resins and balsams) and/or vegetative apices such as bud, leaf primordial and young leaves (Teixeira et al., 2005; Bankova et al., 2019b). The bees use propolis for covering holes and crevices in and around the beehives, maintaining the homeostasis of the hives, and protecting against pests and other invaders. They also use it to expand or reduce ventilation holes in the hive. Moreover, propolis is used as resin reinforcement in the cell structure without which a wild hive would collapse under its own weight (Seeley and Morse, 1976; Simone-Finstrom and Spivak, 2010; Popova et al., 2021c).

In addition to the physical protection it provides, propolis also performs important physiological and biological functions (Simone-Finstrom et al., 2017). Propolis has been shown to be protective against microbial pathogens such as *Paenibacillus larvae*, *Ascosphaera apis*, *Nosema ceranae*, and ectoparasite *Varroa destructor*-associated Deformed Wing Virus in the bee colonies (Mihai et al., 2012; Yemor et al., 2015; Drescher et al., 2017; Simone-Finstrom et al., 2017; Wilson et al., 2017). Moreover, propolis helps maintain social immunity of the colonies by modulating the immune responses. The presence of propolis reduces the bacterial load and consequently lowers the expression immune-related genes such as *hymenoptaecin* and *AmEater*, which are physiologically taxing (Simone et al., 2009). Conversely, propolis increases the expression of immune-related genes such as *defensin-1*, *abaecin*, *hymenoptaecin*, and *apidaecin* in bees when challenged with microbial pathogens (Turcatto et al., 2018).

Propolis is also important in maintaining the antioxidant status and detoxification responses of the bees against environmental stressors such as pesticides and aflatoxins (Niu et al., 2011; Mao et al., 2013). Further confirmation of the importance of propolis in the survival of the honeybees came from Nicodemo et al. (2014) who found that honeybees that were selected for higher propolis production had a longer lifespan and increased brood viability.

Interestingly, many of the therapeutic benefits of propolis within the beehive, such as the antioxidant, immuno-modulator, and antimicrobial properties also apply to human beings and have been demonstrated not only *in vitro* and *in vivo* models, but more importantly in clinical trials (Zulhendri et al., 2021a; 2021b; 2021c, 2022). Silveira et al. (2021) demonstrated that propolis was effective in reducing the symptoms of Covid-19 patients in a randomized placebo-controlled trial. Propolis was also effective in reducing the severity of the symptoms of uncomplicated respiratory-tract infections (Esposito et al., 2021). The immunomodulatory properties of propolis were demonstrated by Conte et al. (2021). They showed that propolis consumption promoted the expression and proliferation of Foxp3 and lymphocyte proliferation in HIV AIDS patients. Moreover, propolis is effective in promoting wound-healing. Afkhamizadeh et al. (2018) found that propolis ointment in addition to the conventional treatments significantly enhanced wound healing based on the decrease in the ulceration area of diabetic foot ulcers, compared to conventional treatments only. Furthermore, Kubat et al. (2021) demonstrated that propolis treatment on wound surfaces following marsupialization of sacrococcygeal pilonidal diseases accelerated wound healing significantly. These clinical trials illustrate the potential of propolis as a health-promoting component for biomaterials.

The chemical composition of propolis varies widely depending upon the geographical location, climate, plant source, and bee species. Propolis generally consists of resin, beeswax, essential oils, pollen, and plant primary and secondary metabolites, such as sugars, amino acids, vitamins, minerals, phenolics, terpenoids, tannins, and alkaloids. However, the bioactive compounds that impart the biological activities of propolis are predominantly thought to be polyphenols and terpenoids (Zulhendri et al., 2021a).

## Orthopedic/Dentistry-Related Biomaterials

Two of the most important aspects to consider with regards to any biomaterial in orthopedic/dental implants are osseointegration and infection prevention (Lu et al., 2021). Any surgical intervention which includes implantation of biomaterials always runs the risks of infections. One of the major hospital-acquired infections is medical device/implant-associated infections, which are usually due to the complex interactions of pathogens, biomaterials, and the immune response of the hosts. In the absence of a foreign body, the infections of the surrounding tissues by the opportunistic pathogens will be cleared by the host’s immune system with relative ease and with lower risks of life-threatening complications. In more severe forms, implant-associated infections can induce local tissue responses where acute and/or chronic inflammation occurs followed by the formation of granulation and fibrous encapsulation. Consequently, the implants become prone to bacterial adhesion, colonization, and infection. The main species of concern are *Staphylococcus aureus*, *Staphylococcus epidermidis*, *Pseudomonas aeruginosa*, and *Enterococcus faecalis*, (Zhang et al., 2014; Cook et al., 2015; Arciola et al., 2018; Drago et al., 2019).

In addition, previous research reported that most bacteria are not free floating but attached to surfaces as biofilms. Biofilms generally consist of bacterial colonies, extracellular polysaccharides, extracellular genetic materials, and (glyco-) proteins. The formation of biofilms greatly enhances the bacterial tolerance to host’s immune systems and/or antimicrobial therapeutics (Arciola et al., 2018; Brum et al., 2020). Biofilm formation has been directly and indirectly implicated in implant failures (Daubert and Weinstein, 2019). Therefore, its management is paramount in biomaterial implants. Propolis has been shown to be effective in reducing biofilm formation. Propolis works through several mechanisms of actions such as by affecting the swimming and swarming motility of microbes, modifying the architecture of the biofilms, and inhibiting quorum sensing (De Marco et al., 2017; Veloz et al., 2019; Popova et al., 2021a).

Another issue to be addressed when biomaterials are used for implants is osseointegration. As well as needing to be biocompatible, i.e. not triggering significant immune response or foreign-body response, biomaterials need to be able to promote the adherence and proliferation of osteoblasts on the surface of the implants. Moreover, biomaterials also need to be able to recruit stem cells from the surrounding tissues and subsequently promote the differentiation of those stem cells into osteogenic cells (Zhang et al., 2014; Choi and Park, 2018).

Due to its strong antimicrobial, antioxidant, and immune-modulatory properties, propolis potentially can play an important role either as a coating material or a component in the implant itself. Propolis extracts from various geographical regions: Europe, Asia, Africa and the Americas, have been shown to have strong antimicrobial properties against *S. aureus*, *S. epidermis*, *P. aeruginosa*, and *E. faecalis* (Przybyłek and Karpiński, 2019). It is postulated that propolis works by exerting direct antimicrobial activity through disrupting the permeability of the bacterial cellular membrane and therefore damaging its membrane potential and ATP generation ability. In addition, propolis also works through immunomodulatory pathways by stimulating the host’s natural defense/immune system (Zulhendri et al., 2021a).

Additionally, the immunomodulatory and anti-inflammatory properties of propolis make it a suitable bioactive component in biomaterials. The anti-inflammatory properties of propolis appear to be conserved regardless of the geographical sources of propolis. M Sahlan et al. (2021) demonstrated that Indonesian propolis extracts significantly reduced the expression of tumor necrosis factor (TNF)-α, inducible nitric oxide synthase (iNOS) and nitric oxide (NO) in lipolysaccharide (LPS)-induced macrophages. Batista et al. (2018) found that the antioxidant and anti-inflammatory properties of Brazilian red propolis extract significantly protected the UVB-induced skin damage in murine models. In addition, Touzani et al. (2019) investigated the effect of propolis extract from the northern Morocco region and demonstrated its anti-inflammatory effect in LPS-stimulated human peripheral blood mononuclear cells. Propolis extracts significantly reduced the expression of inflammatory TNF-α and interleukin-6 (IL-6) and increased the expression of the anti-inflammatory IL-10 in a dose-dependent manner. Furthermore, Jalali et al. (2020) and Shang et al. (2020) carried out systematic reviews and meta-analyses of clinical trials investigating the effect of propolis consumption on inflammatory markers. They independently identified six clinical trials and found a significant reduction in inflammatory markers interleukin-6 (IL-6), C-reactive protein (CRP), and TNF-α following propolis consumption. More importantly, these clinical trials carried out on propolis sourced from various regions, namely Iran, Brazil, Chile, Denmark, China, and Uruguay, confirmed the conserved anti-inflammatory properties of propolis (Jalali et al., 2020; Shang et al., 2020).

### Hydroxyapatite/Calcium Phosphate-Based Biomaterials

Hydroxyapatite, apart from being the main component of mammalian bones, is also a chemically and thermally stable form of calcium phosphate and considered one of the most widely applied biomaterials for medical applications especially for orthopedic/dentistry-related applications (Prakasam et al., 2015). Several studies have explored the potential of combining propolis with hydroxyapatite as a mean of increasing its potential as a biomaterial. Grenho et al. (2015) investigated the effect of Brazilian red and green propolis extracts on nanohydroxyapatite (nano-HA) biomaterials. Nano-HA materials were immersed in propolis-containing solutions. The resulting nano-HA-propolis composites were found to have reduced hydrophilicity. In addition, the nano-HA-propolis biomaterials were also challenged with *S. aureus*. It was found that the nano-HA impregnated with the highest concentration of propolis tested (25 μg/ml) had 99% reduction in viable bacteria. Furthermore, the propolis-incorporated nano-HA did not exhibit cytotoxicity. More importantly, an increase in cellular metabolic activity of murine fibroblast cells in the presence of the propolis-treated nano-HA was observed when compared to the non-treated-nano HA (Grenho et al., 2015).

Wijayanti et al. (2020) also demonstrated that Brazilian propolis extract incorporated with carbonated hydroxyapatite induced the growth of NIH 3T3 fibroblast cells. The highest concentration tested (10% wt propolis extract) appeared to have the highest growth level, up to ∼125% growth compared to controls. On the other hand, Scatolini et al. (2018) showed that Brazilian red and green propolis extracts-incorporated with hydroxyapatite appeared to exhibit cytotoxicity against CHO-k1 cell lines. They also found that the propolis extracts reduced the degree of agglomeration of the hydroxyapatite suggesting the negative effect of the propolis extracts on the physicochemical properties of the biomaterials. On a positive note, they demonstrated the antimicrobial activity of propolis extracts-incorporated hydroxyapatite against *S. aureus*. Interestingly, propolis was shown to reduce osteoclastogenesis by modulating RANKL expression. Devitaningtyas et al. (2020) found that the RANKL expression in rabbit’s alveolar bones was reduced by the propolis-incorporated with carbonated hydroxyapatite.

Furthermore, propolis can complement the function of Casein Phosphopeptide- Amorphous Calcium Phosphate (CPP-ACP) by providing superior antimicrobial properties. ACP-CCP is used for remineralizing tooth surfaces (Chhabra and Chhabra, 2018). Soekanto et al. (2017) and Hasnamudhia et al. (2017) demonstrated that propolis enhanced the antimicrobial activity against *Streptococcus mutans*. They also found superior release of calcium and phosphate ions in a chewing gum formulation containing CPP-ACP and propolis, compared to CPP-ACP only controls. Muhamad Sahlan & Purwanti (2016) found that propolis helped CCP-ACP to bind more uniformly onto the surface of demineralized enamel. They postulated it was due to the occlusive effect of the polyphenols in the propolis extracts. This effect was also confirmed by Amalina et al. (2017). They observed that without the presence of propolis, the CCP-ACP was deposited unequally on the surface of the enamel. The problem was ameliorated by the incorporation of propolis into the CCP-ACP formulation. These studies illustrate the potential of propolis in enhancing the functional properties of calcium phosphate-based biomaterials, not only through its biological activities but also in terms of the physicochemical modification and/or enhancement.

### Glass/Bioglass-Related Biomaterials

Bioglass or bioactive glass is a biomaterial that has several key properties; an amorphous structure, a glass-transition-range (Tg) temperature behavior, and is composed of two common structures, i.e. network formers and network modifiers. Network formers are compounds that can form glass structures which are mainly silica (SiO_2_), phosphorus pentoxide (P_2_O_5_), and boron trioxide (B_2_O_3_). Network modifiers are compounds that can modify the glass structure by altering the bridging oxygen atoms into non-bridging ones. Modifiers are usually oxides of alkali or alkaline-earth metals, such as sodium, calcium, and strontium. Hence, bioactive glasses are usually categorized into three classes: silicate-based, phosphate-based, and borate-based (Brauer, 2015; Rahaman et al., 2018; Li et al., 2019).

Several studies investigated the effect of propolis on glass ionomer cements (GICs) for dental applications. Ethanolic Brazilian green propolis extracts were found to increase water sorption of the Ketac Fil Plus, ChemFlex and Ketac Molar Easymix GICs. However, one GIC (ChemFlex) was negatively impacted in terms of diametral tensile strength by the incorporation of propolis extracts (Troca et al., 2011). In addition, propolis also appears to increase water solubility of the GICs and significantly reduces the compressive strength. Subramaniam et al. (2017) showed that the addition of 1% w/v of propolis extract was enough to reduce the compressive strength of GICs by ∼7.5%. In addition, Panahandeh et al. (2021) explored the compatibility of 25% and 50% (w/w) propolis extract with GICs. It was found that the flexural and shear bond strength were inversely proportionate to the concentration of propolis in the composites. The 50% propolis-GIC composites had the lowest flexural and shear bond strength whereas the control group (GIC only) had the highest flexural and shear bond strength.

Conversely, (Hatunoǧlu et al., 2014), did not observe any detrimental effect of propolis on shear-peel band strength of the GICs. On the other hand, propolis appeared to increase the microhardness measured with Vickers hardness of the GICs and did not affect the performance of the GICs in terms of microleakage (Altunsoy et al., 2016). Prabhakar et al. (2016) also did not observe any detrimental effect of the addition of 1% v/v propolis extract in terms of shear bond strength of GICs. Moreover, fluoride release appeared to be enhanced by the addition of propolis (Prabhakar et al., 2016; Elgamily et al., 2018). Galarraga-Vinueza et al. (2018) investigated the effect of propolis embedding on the physicochemical properties of 58S mesoporous bioactive glass (MBG) particles and found that propolis did not hinder the ability of MBGs in forming the hydroxyapatite layer, which is crucial in regeneration applications.

Furthermore, Andrade et al. (2019a) showed that the incorporation of propolis extract (37% w/w) to the GICs increased the mechanical properties and thermal stability of GICs, when compared to propolis free-GICs. It was observed that the incorporation of propolis extract increased the compressive strength of GIC by ∼25% (from ∼173 to 217 MPa). The improvement in the physical properties appeared to be related to the modification of GIC microstructures by the propolis extract (Andrade et al., 2019b). In addition, Meneses et al. (2020) investigated the effect of increasing concentration of propolis (10–50% w/w) on two types of glass ionomers (Meron and Ketac Cem) in animal models. They found that the propolis did not have any negative impact on the physical properties of the GICs in terms of shear bond strength test (SBST) and adhesive remnant index (ARI). They observed an inflammatory response marked by the presence of multinucleated giant cells and CD68^+^ for macrophages on tissues that were treated with the 10% propolis-GIC composites. However, this inflammatory response was absent in the tissues treated with the composites with higher propolis concentrations. It was postulated that the inflammatory response was driven by the relatively higher ethanol concentration in the 10% propolis-GIC composites. The overall trend observed in the study was that the inflammatory histological changes were inversely correlated with the concentrations of the propolis in propolis-GIC composites (Meneses et al., 2020; De Meneses et al., 2021).

One of the main advantages of the incorporation of propolis into the glass biomaterials is the antimicrobial property enhancement. For example, by incorporating the Turkish propolis into GICs clearly exhibited superior antimicrobial and anti-biofilm activities against *S. mutans* (Topcuoglu et al., 2012). Elgamily et al. (2018) also demonstrated that the antimicrobial activity of propolis in GICs. The antimicrobial activity of propolis extract-glass ionomers composites appears to correlate with the flavonoids content (Andrade et al., 2019a). Andrade et al. (2019b) demonstrated that the propolis had synergistic effect with GICs against several oral pathogens; *S. mutans*, *S. salivarius*, C. *albicans*. Perhaps unsurprisingly, Meneses et al. (2020) and Saputra et al. (2020) showed the antimicrobial activity of propolis-glass ionomer composites was dependent upon the concentration of the incorporated propolis.

These studies illustrate the heterogeneity of results in terms of physicochemical and functional properties of propolis-GIC composites. Several factors could contribute to the lack of uniform results. We observed that the types and brands of GICs play a major role in determining the suitability and compatibility of the final composite products. In addition, the types of propolis extracts also appear to be a contributing factor. Optimization studies with regards to the concentrations of propolis extracts to be incorporated need to be extensively explored. Moreover, propolis extracts are known for their complexity in terms of the composition of the chemical compounds which are dependent on their geographical source, type of bees, and type of extract. Therefore, more studies are needed to standardize the propolis-GIC composites prior to their adoption in the clinical settings.

### Titanium

Titanium and its alloys have been widely considered and used as medical and dental implant devices due to their corrosion resistance, strength, and high performance in terms of compatibility. One of the main disadvantages of metals, including titanium, is that they do not have biological functions. Therefore, surface modifications and/or treatments are often necessary to promote biocompatibility and functionalities (Hanawa, 2019; Kaur and Singh, 2019). Propolis with its antimicrobial and anti-inflammatory properties could potentially be a useful substance to overcome problems associated with titanium and its alloys.

There are still a very limited number of studies investigating the effect of propolis on titanium-based biomaterials. Martorano-Fernandes et al. (2020) investigated the fungicidal effect of propolis against *C. albicans* biofilm on titanium surfaces. It was found that 3% (w/v) propolis extract had antifungal activity, comparable to standard treatment of 0.12% (v/v) chlorhexidine, against monoculture *C. albicans* biofilm on the titanium surfaces. However, the fungicidal effect of propolis was inferior, compared to 0.12% (v/v) chlorhexidine, when the titanium surfaces were colonized by co-culture of *C. albicans* and *C. glabrata* biofilms. This suggests the concentration of propolis could be increased to enhance its antifungal activity.

Predictably, propolis appears to improve the corrosion resistance of titanium as it is well known for its antioxidant properties (Martinello and Mutinelli, 2021). Kakaa et al. (2020) demonstrated the effect of propolis in preventing corrosion on commercially pure titanium (cp-Ti grade 2) that was exposed to artificial fluoride saliva. After 72 h of exposure to artificial saliva, corrosion was observed through the occurrence of roughness on the surface of the titanium discs and also the detection of TiO_2_ layer. On the other hand, the corrosion on the titanium discs treated with propolis was undetected and propolis was observed to coat the surface of the titanium discs evenly.

Moreover, TiO_2_ nanotubes generated by anodizing titanium are a promising biomaterial which has superior interaction with osteoblasts, compared to the biologically inert titanium, in promoting bone-implant integration (Brammer et al., 2012). Somsanith et al. (2018) investigated the compatibility of propolis in TiO_2_ nanotubes for dental implants in animal models (rat mandibles). It was observed from the histological analyses; micro-computed tomography (*µ*-CT) and hematoxylin and eosin (HE) staining that the bone formation and mineral density were significantly higher in animals that were implanted with propolis-TiO_2_ nanotubes. After 4 weeks, the bone mineral density and new bone volume were on average ∼15% and ∼27% higher, respectively in rats implanted with propolis-TiO_2_ nanotubes compared to TiO_2_ nanotubes alone. The expression of bone formation molecules BMP-2 and seven was also notably higher around the propolis-TiO_2_ nanotubes. Furthermore, the expression of inflammatory cytokines IL-1β and TNF-α was higher around the surface of the TiO_2_ nanotubes compared to propolis- TiO_2_ nanotubes, illustrating the anti-inflammatory properties of propolis. Table 1 summarizes the potential use of propolis in orthopedic/dentistry-related biomaterials.

**TABLE 1:** Summarizes the potential use of propolis in orthopedic/dentistry-related biomaterials. This table should be considered as examples and by no means exhaustive.

Types of Biomaterials	Measured Outcome	References
Hydroxyapatite/Calcium Phosphate-Related Biomaterials		
Nanohydroxyapatite-propolis	Reduced hydrophilicity. Antimicrobial activity against *S.aureus.* Not cytotoxic	Grenho et al. (2015)
Carbonated hydroxyapatite- propolis	Not cytotoxic. Induced the growth of NIH 3T3 fibroblast cells	Wijayanti et al. (2020)
Hydroxyapatite-propolis	Cytotoxicity against CHO-k1 cell line. Reduction in the degree of agglomeration of hydroxyapatite. Antimicrobial activity against *S.aureus*	Scatolini et al. (2018)
Carbonated hydroxyapatite-propolis	Reduction in RANKL expression	Devitaningtyas et al. (2020)
CPP-ACP-propolis	Antimicrobial activity against *Streptococcus mutans*. Superior release of calcium and phosphate ions	Soekanto et al. (2017), Hasnamudhia et al. (2017)
CPP-ACP-propolis	Propolis helped CCP-ACP to be bound more uniformly on the surface of demineralized enamel	(Sahlan and Purwanti, 2016) (Amalina et al., 2017)
Glass/bioglass-related biomaterials		
Glass ionomer cements (GICs)-propolis	Potential reduction in diametral tensile strength and compressive strength	Troca et al. (2011); Subramaniam et al. (2017)
	Reduction in flexural and shear bond strengths	Panahandeh et al. (2021)
	Propolis did not affect the shear-peel band strength of GICs. Antimicrobial activity against *S.mutans*	Hatunoǧ;lu et al. (2014)
	Propolis increased the microhardness of the GICs and did not affect the performance of the GICs in terms of microleakage	Altunsoy et al. (2016)
	Shear bond strength of GICs was not affected by propolis. Enhancement of fluoride release. Antimicrobial properties	Prabhakar et al. (2016); (Elgamily et al. (2018)
	Propolis increased the mechanical properties, thermal stability, and compressive strength of GICs. Antimicrobial properties	Andrade et al. (2019b)
	Anti-inflammatory effect of propolis. Antimicrobial properties	Meneses et al. (2020); De Meneses et al. (2021)
	Antimicrobial properties	Topcuoglu et al. (2012)
58S mesoporous bioactive glass (MBG)-propolis	Propolis did not hinder the ability of MBGs in forming the hydroxyapatite layer	Galarraga-Vinueza et al. (2018)
Titanium		
Titanium-propolis	Antifungal effect against *C.albicans*	Martorano-Fernandes et al. (2020)
	Corrosion inhibitor	Kakaa et al. (2020)
TiO_2_- propolis	Anti-inflammatory properties. Upregulated bone formation *in vivo*	Somsanith et al. (2018)

## Skin Dressing-Related Biomaterials

Wound management has always been an important challenge in global medical systems. In addition, mortality from chronic wounds is even said to be comparable to cancers (Las Heras et al., 2020). Challenges such as limitations in autologous tissues and skin donors and the severity and extent of injuries, damage, and scar contracture in the area of the skin grafts, further exacerbate and complicate the issue (Peng et al., 2022). Propolis has been shown to have wound-healing and antimicrobial properties which make it a suitable component to be incorporated into biomaterials for wound and/or skin dressing purposes (da Silva et al., 2020).

Aqueous propolis extract was demonstrated to not negatively influence the structure of electrospun polyvinyl alcohol (PVA) fibre mats. The analysis of release kinetics of the phenolics showed that 86–96% of vanilic acid, caffeic acid, vanillin acid, *p*-coumaric acid and ferulic acid in the propolis extract was released from the PVA fibers after 15 min, demonstrating the suitability of propolis-PVA fiber mats for targeted delivery biomaterials (Adomaviciute et al., 2015). The same group also demonstrated the synergistic effect of propolis and silver nanoparticles in the electrospun polyvinylpyrrolidone (PVP) fiber mats against *S. aureus*, *S. epidermidis*, *E. faecalis*, *E. coli*, *P. aeruginosa*, *P. vulgaris*, *B. subtilis*, *B. cereus*, and *C. albicans*. It was shown that the combination of 6% wt propolis and 10% wt Ag in colloidal solution had the best release profile of bioactive compounds (Adomaviciute et al., 2016).

In addition, Sutjarittangtham et al. (2015) investigated the antibacterial and antiviral properties of PVA (top and base layers) and PVP (middle layer) fiber mats containing propolis with or without cross-linked carboxymethyl starch (CL-CMS) against *S. aureus,* methicillin-resistant *S. aureus* (MRSA), *P. aeruginosa B. cereus*, *S. epidermidis,* HSV-1F, and HSV-2G, The carboxymethyl starch was used as a swelling agent. It was found that the antibacterial and antiviral activities of the fiber mats were dose-dependent on propolis concentration and the CL-CMS did not have any effect on the antimicrobial activity.

Furthermore, natural rubber latex (NRL) is a tissue-compatible biomaterial suitable for wound/skin dressing that can be utilized to deliver bioactive compounds (Balabanian et al., 2006). Zancanela et al. (2017) demonstrated that no chemical interaction occurred between hydro-ethanolic extract of propolis and NRL. The propolis-NRL composite demonstrated antifungal activity against *C. albicans*. Predictably, the antifungal activity correlated with the amount of propolis extract that was released from the biomaterial. Nevertheless, it appears the bioactive compound release was considerably more gradual compared to other biomaterials discussed in this review. Thirty seven percent of the propolis extract was released in 5 h and it reached a plateau at ∼150 h with ∼55% of the propolis extract being released. It was noted that the propolis incorporation into NRL improved the mechanical properties of NRL by ∼75%, ∼23%, and ∼53% in terms of tensile strength, percentage of “stretch at break”, and Young’s modulus, respectively (Zancanela et al., 2017). In addition, they also compared the performance of NRL incorporated with three different types of propolis extract; green, red, and poplar propolis (Zancanela et al., 2019). In terms of the release kinetic of the propolis extracts, they were similar in the first 5 h. However, the release kinetics became significantly different later. NRL-green propolis had the highest bioactive compound release followed by poplar- and red propolis-NRL composites. Interestingly, it was found that the green propolis-NRL composite had the lowest antifungal activity against *C. albicans* and it was postulated that some bioactive compounds of the green propolis were not released from the NRL matrix. In addition, the NRL-green propolis extract also had the lowest mechanical strength. Furthermore, they also investigated the cytotoxicity of the NRL-propolis composites against 3T3 fibroblast cells and found that at 30% (wt) propolis, none of the composites were cytotoxic. However, at 50% propolis extract, the NRL-green propolis became cytotoxic (Zancanela et al., 2019). This study illustrates the challenges posed by propolis extracts due to their complexity and heterogeneity in terms of the content of the bioactive compounds in various types of propolis.

Furthermore, Krupp et al. (2019) investigated the compatibility of propolis extract harvested from the beehives of the stingless bee *Scaptotrigona polysticta* as the bioactive substance in the NRL membrane for alleviating second-degree burns in animal models. It was found, based on the histopathological assessment, that the propolis enriched-NRL membrane treated animals had a lower number of inflammatory infiltrates and/or cells in the epithelial area. They also observed the formation of keratinocytes in the epidermal layer which consequently allowed the detachment of the formed crust from the necrotic/damaged tissues. The formation of new blood vessels and tissue reorganization also occurred earlier in the animals treated with propolis-NRL membrane. The wound-healing effect of propolis-NRL membrane was comparable to silver sulfadiazine (Krupp et al., 2019).

Several studies explored the potential and compatibility of propolis with corn-derived biomaterials such as zein and cornstarch. Eskandarinia et al. (2019) investigated the antimicrobial activity and biocompatibility of cornstarch-hyaluronic acid-propolis film for wound dressing and healing purposes. It was demonstrated that the antimicrobial properties against *S. aureus*, *E. coli,* and *S. epidermidis* were correlated with propolis concentration. No cytotoxicity was observed against L929 fibroblast cells. The wound healing efficacy of the cornstarch-hyaluronic acid-propolis composite film was also tested against cornstarch only and cornstarch-hyaluronic acid films on animal models. The former clearly accelerated wound healing/closure on the animals compared to the latter two, measured by the changes in wound area during the 14 days observation period (Eskandarinia et al., 2019). In addition, electrospun zein (corn-derived water insoluble protein) and propolis fiber mats were also demonstrated to be a viable wound-healing biomaterial (Moradkhannejhad et al., 2018). The zein-propolis composite fibers had good antimicrobial activity against *E. coli*, *S. enterica*, *P. aeruginosa* and *C. albicans*. The addition of propolis into zein fibre mats appeared to increase the fibre size from 264 to 419 nm. However, no physicochemical implications were explored in this study (Moradkhannejhad et al., 2018). Other materials such as polyurethane, cellulose, chitosan, and silk have also been shown to be compatible with propolis (Voss et al., 2018; Khodabakhshi et al., 2019; Khoshnevisan et al., 2019; Baygar, 2020; Ceylan, 2021; Ionescu et al., 2021; Sharaf et al., 2021). Table 2 summarizes the potential use of propolis in skin/wound dressing-related biomaterials.

**TABLE 2:** Summarizes the potential use of propolis in skin/wound dressing-related biomaterials. This table should be considered as examples and by no means exhaustive.

Types of Biomaterials	Measured Outcome	References
Polyvinyl alcohol (PVA)/polyvinylpyrrolidone (PVP)-propolis	Kinetic release of phenolics reached 86–96%. Synergism with silver nanoparticles	Adomaviciute et al. (2015); Adomaviciute et al. (2016)
Polyvinyl alcohol (PVA)/polyvinylpyrrolidone (PVP)- cross-linked carboxymethyl starch (CL-CMS)-propolis	Antimicrobial properties against *S. aureus,* methicillin-resistant *S. aureus* (MRSA), *P. aeruginosa B. cereus*, *S. epidermidis,* HSV-1F, and HSV-2G	Sutjarittangtham et al. (2015)
Genipin-crosslinked PVA/chitosan-propolis	Promoted cell proliferation suitable for wound healing application	Ceylan, (2021)
poly-ε-caprolactone (PCL)/chitosan electrospun mat on polyurethane/propolis	Antimicrobial properties and better wound healing properties	(Karizmeh et al., 2022)
Natural rubber latex (NRL)-propolis	Antifungal activity against *C.albicans.* Improvement in mechanical properties. Cytotoxicity was observed in NRL-green propolis composite	(Zancanela et al., 2017, 2019)
	Anti-inflammatory properties. Wound healing properties *in vivo*	Krupp et al. (2019)
Cornstarch-hyaluronic acid-propolis	Antimicrobial properties against *S. aureus*, *E. coli,* and *S. epidermidis.* Accelerated wound healing *in vivo*	Eskandarinia et al. (2019)
Zein-propolis	Antimicrobial activity against *E. coli*, *S. enterica*, *P. aeruginosa* and *C. albicans*	Moradkhannejhad et al. (2018)
Silk-silver nanoparticles-propolis	Antimicrobial properties against *E. coli* and *S. aureus* No cytotoxicity detected	Baygar, (2020)
Polyurethane-propolis	Propolis decreased the mechanical properties of polyurethane in terms of tensile, contact angle, and water absorption. Propolis increased the “elongation at break”. Propolis increased the antimicrobial properties and cellular compatibility	Khodabakhshi et al. (2019)

## General Discussion and Future Directions

The regulatory guidelines for the medical devices have changed significantly in the recent times. Hence, the lack of robust and consistent quality and *in-vivo* data limits the translation of the research to the commercial level. Rohde and Hesselbarth (2005) applied for a patent for the use of propolis and its bioactive compounds, especially caffeic acid phenethyl ester (CAPE) for coated medical implants such as stent, pacemaker, cardiac or venous valve and vascular prosthesis for restenosis prophylaxis after percutaneous transluminarcoronary angioplasty (PTCA). The patent was filed based on the *in-vitro* studies including vitality test, proliferation test and NF-κB activation using the human arterial endothelial cells (ECs) and Smooth Muscle Cells (SMCs). The vitality of both cells was dependent on the concentration of propolis though significantly higher sensitivity was observed for the SMCs compared to ECs. Similarly, the proliferation of both the cells increased with the increase in the concentration of propolis. However, there was no animal or human trial data reported using propolis coated implants in the patent document.

The main drawback of propolis as a bioactive component of biomaterials is the heterogeneity and/or complexity of the chemical compositions and consequently the difficulty in standardizing the extracts. The types of extracts, such as ethanolic, methanolic, hydroethanolic, and glyceric extracts, and so on and methods for extracting the bioactive compounds will also influence the biological and physicochemical properties of the resulting extracts. In addition, the majority of the studies explored in the review are still in the *in-vitro* stage coupled with limited number of *in-vivo* studies.

**FIGURE 1:**
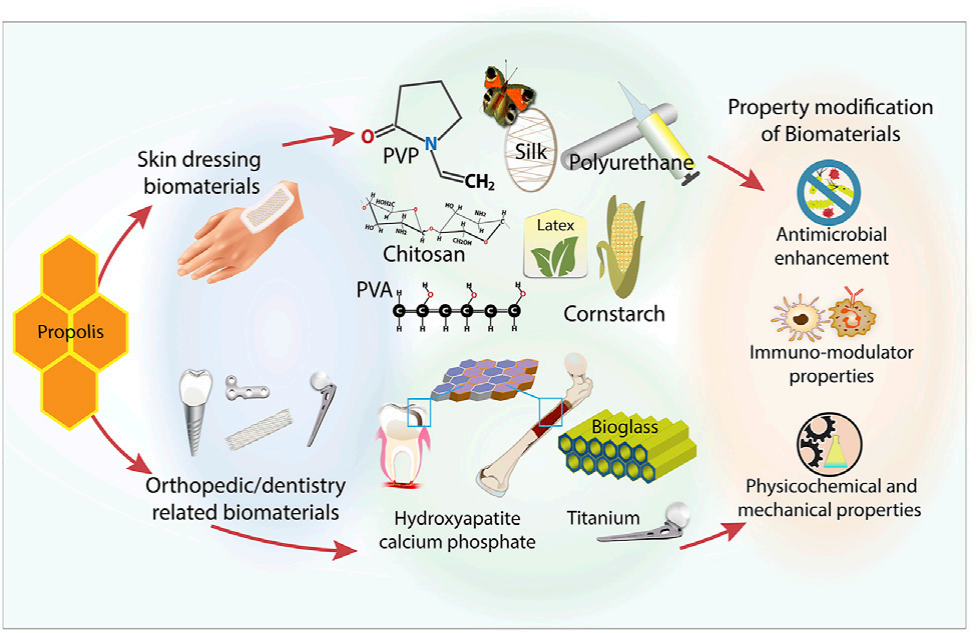
The potential role of propolis as a component of biomaterials.

The studies analyzed and presented in this review illustrate the suitability and compatibility of propolis as a bioactive component in biomaterials. The efficacy and compabitility of propolis depend upon factors, such as types of extracts and types of biomaterials. Figure 1 summarizes the potential role of propolis in biomaterials. Generally, propolis appears to be compatible with hydroxyapatite/calcium phosphate-based biomaterials. Propolis enhances the antimicrobial properties of the resulting composite materials. In some studies, propolis improves the physicochemical properties of hydroxyapatite/calcium phosphate-based biomaterials, such as in terms of the release of calcium and phosphate ions and its ability to bind more uniformly to enamel.

However, the effect of propolis on the glass-based biomaterials cannot be generalized. Depending on the concentration, types of extract, and geographical sources of the propolis, the effect on the glass biomaterials can either be an improvement or detrimental in terms of mechanical properties such as compressive strength and shear bond strength. In addition, there is a limited data on the effect propolis on titanium-based biomaterials. Propolis may be considered to be most compatible with wound/skin dressing biomaterials. The majority of the studies in this area found no significant negative effects of propolis on the physicochemical properties of the composite biomaterials for skin/wound dressings. Two of the more consistent impacts of propolis across these different types of biomaterials are the enhancement of the antimicrobial and the immune-modulator/anti-inflammatory properties resulting from the combination of propolis and biomaterials.
